# Epidermal keratinocytes initiate wound healing and pro-inflammatory immune responses following percutaneous schistosome infection

**DOI:** 10.1016/j.ijpara.2014.11.002

**Published:** 2015-03

**Authors:** Claire D. Bourke, Catriona T. Prendergast, David E. Sanin, Tate E. Oulton, Rebecca J. Hall, Adrian P. Mountford

**Affiliations:** Centre for Immunology and Infection, University of York, York YO10 5DD, United Kingdom

**Keywords:** Keratinocyte, Skin, Schistosome, Wound healing, Immune response, Epidermis

## Abstract

Keratinocytes constitute the majority of cells in the skin’s epidermis, the first line of defence against percutaneous pathogens. Schistosome larvae (cercariae) actively penetrate the epidermis to establish infection, however the response of keratinocytes to invading cercariae has not been investigated. Here we address the hypothesis that cercariae activate epidermal keratinocytes to promote the development of a pro-inflammatory immune response in the skin. C57BL/6 mice were exposed to *Schistosoma mansoni* cercariae via each pinna and non-haematopoietic cells isolated from epidermal tissue were characterised for the presence of different keratinocyte sub-sets at 6, 24 and 96 h p.i. We identified an expansion of epidermal keratinocyte precursors (CD45^−^, CD326^−^, CD34^+^) within 24 h of infection relative to naïve animals. Following infection, cells within the precursor population displayed a more differentiated phenotype (α6integrin^−^) than in uninfected skin. Parallel immunohistochemical analysis of pinnae cryosections showed that this expansion corresponded to an increase in the intensity of CD34 staining, specifically in the basal bulge region of hair follicles of infected mice, and a higher frequency of keratinocyte Ki67^+^ nuclei in both the hair follicle and interfollicular epidermis. Expression of pro-inflammatory cytokine and stress-associated keratin 6b genes was also transiently upregulated in the epidermal tissue of infected mice. In vitro exposure of keratinocyte precursors isolated from neonatal mouse skin to excretory/secretory antigens released by penetrating cercariae elicited IL-1α and IL-1β production, supporting a role for keratinocyte precursors in initiating cutaneous inflammatory immune responses. Together, these observations indicate that *S.mansoni* cercariae and their excretory/secretory products act directly upon epidermal keratinocytes, which respond by initiating barrier repair and pro-inflammatory mechanisms similar to those observed in epidermal wound healing.

## Highlights

• A murine model of cutaneous schistosome infection shows activation of the epidermis.
• Epidermal keratinocyte precursor cells in the hair follicle expand after infection.
• Infection leads to increased expression of pro-inflammatory genes in the epidermis.
• Keratinocytes exposed to schistosome antigens in vitro secrete IL-1α and IL-1β.
• Keratinocyte responses to schistosome infection and antigens mimic wound healing.

## Graphical abstract

**Figure:**
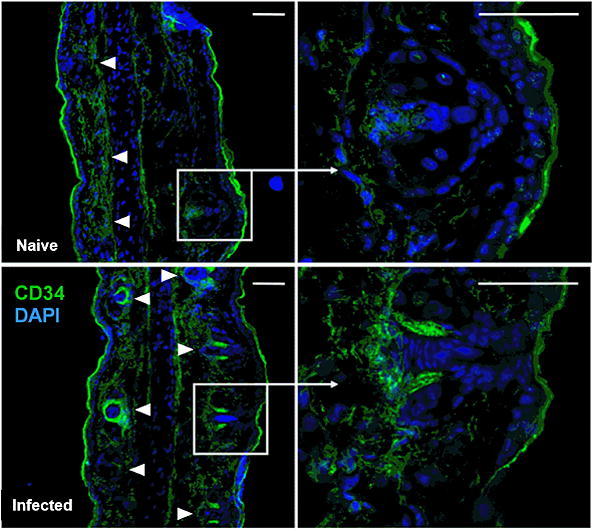

## 1 Introduction

Schistosomes are a major parasite of humans currently infecting over 230 million people world-wide (World Health Organization (WHO), 2012, http://www.who.int/mediacentre/factsheets/fs115/en/) and causing a debilitating chronic disease (schistosomiasis) responsible for an estimated 70 million disability-adjusted life years per annum (King et al., 2005; King and Dangerfield-Cha, 2008). The larval stage (cercariae) of the schistosome life-cycle is the first to interact with the host and actively invades the skin via secretion of excretory/secretory (E/S) antigens (Mountford and Trottein, 2004; Jenkins et al., 2005b; Paveley et al., 2009), including proteolytic enzymes and immunogenic glycans (McKerrow et al., 1985; Knudsen et al., 2005; Curwen et al., 2006; Harn et al., 2009; Paveley et al., 2011). Following initial invasion most *Schistosoma mansoni* cercariae mature into schistosomula and reside in murine skin for at least 2 days, during which time the epidermal basement membrane provides a temporary barrier to onward migration (Wheater and Wilson, 1979). Following arrival in the dermis, schistosomula seek a blood vessel in order to exit the skin via an intravascular route, migrate via the lungs and mature into the adult stage of their life cycle in the hepatic portal system (Wheater and Wilson, 1979; Jenkins et al., 2005b).

Schistosome cercariae are known to affect the function of dermal and epidermal antigen presenting cells (APCs) of the innate immune system (e.g. Langerhans cells (LCs) and dendritic cells (DCs) (Angeli et al., 2001; Kumkate et al., 2007; Cook et al., 2011)). However, the role of non-professional immune cells such as epidermal keratinocytes has not been investigated in the context of cutaneous schistosomiasis. Keratinocytes are of particular interest since they constitute the majority of cells in the skin’s primary barrier against invading pathogens, the epidermis (Wood et al., 1992; Martin, 1997; Madison, 2003; Pivarcsi et al., 2003), and are likely to be the first cell type exposed to *Schistosoma* cercariae and their E/S antigens. In addition to providing a physical barrier to infection, keratinocytes mediate skin homeostasis (Blanpain and Fuchs, 2009; Nagao et al., 2012) and respond rapidly to mechanical insult via secreting soluble factors (e.g. cytokines and chemokines (Hong et al., 2001; Nagao et al., 2012)) and increasing their proliferative responses in order to restore damaged tissue (Trempus et al., 2003; Smithgall et al., 2008; Gause et al., 2013). Keratinocytes are also known to be a source of stress-associated cytokines produced in response to a number of other cutaneous pathogens (e.g. *Candida albicans* (Wollina et al., 2004), *Trichobilharzia* spp. (Ramaswamy et al., 1995b) and *Sarcoptes scabiei* (Mullins et al., 2009)). Observations in mechanical wounding models suggest that phenotypic diversity between epidermal keratinocytes may also influence the development of such responses (Jensen et al., 2008; Blanpain and Fuchs, 2009; Nagao et al., 2012; Plikus et al., 2012). For example, keratinocytes located in different epidermal niches, particularly within hair follicle structures, display distinct chemokine repertoires involved in recruitment of epidermal LC precursors (Nagao et al., 2012). Thus both the nature of keratinocyte responses and their location within the epidermis may contribute to the initiation of immune responses to invading cercariae at the site of infection.

Unlike the larvae of *Schistosoma* spp. that infect birds, which elicit dermatitis at their point of entry in mammalian skin (Kourilova et al., 2004), a single percutaneous exposure to *S. mansoni* cercariae does not result in an overt tissue lesion in murine infection models. However, repeated exposure to *S. mansoni* cercariae causes more tissue damage than a single exposure (Cook et al., 2011) and promotes both angiogenic responses, (i.e. formation of new blood vessels from existing vessels) also active during wound-healing (Aynsley, S.A. 2011. Exploring the dermal immune and angiogenic responses to *Schistosoma mansoni*. PhD thesis, University of York, UK.), and a CD4+ T helper (Th) 2 polarised immune response (Cook et al., 2011). Identification of commonality between the Th2-type immune responses to helminth parasites, including schistosomes (Cook et al., 2011), and those involved in tissue repair suggest that there is cross-talk between the two pathways in affected tissues (Gause et al., 2013). Epidermis-derived alarmins such as IL-33 and thymic stromal lymphopoietin (TSLP), and the innate pro-inflammatory mediators IL-1α and IL-1β are involved at an early stage in these pathways and constitute one route by which physically restricted keratinocytes in the epidermis may influence infiltrating APCs and dermal stroma (Maas-Szabowski et al., 2000; Smithgall et al., 2008; Ramalingam et al., 2009; Gause et al., 2013). Thus, changes in epidermal keratinocyte subtypes may have important consequences for the subsequent activation and conditioning of acquired immune responses to larval schistosomula and tissue damage caused by parasite migration.

In this study, we address the hypothesis that percutaneous invasion by *S. mansoni* cercariae elicits changes in epidermal keratinocyte populations per se, as well as influencing expression of pro-inflammatory cytokines and alarmins in whole epidermal tissue. Since keratinocytes are in close contact with epidermal leukocytes (e.g. LC and γδ T cells), we investigated whether cercarial E/S antigens directly affect keratinocyte production of the cytokines IL-1α, IL-1β and TSLP, using an in vitro model of basal keratinocyte precursors grown in the absence of other cell types (Caldelari et al., 2000; Lichti et al., 2008). We believe that our observations provide the first indication that schistosome cercariae and their E/S products act directly upon epidermal keratinocytes, which respond by initiating barrier repair and pro-inflammatory mechanisms.

## 2 Materials and methods

### 2.1 Ethics statement

All experimental procedures involving animals were conducted in accordance with the United Kingdom Home Office Animals (Scientific Procedures) Act of 1986 and were approved by the University of York, UK, Ethics Committee.

### 2.2 *S. mansoni* parasites and percutaneous infection

Anaesthetised 6–10 week old female C57BL/6 mice were exposed via each pinna to 200 *S. mansoni* cercariae (Puerto Rican strain) for 20 min to allow percutaneous infection to occur (detailed method reported elsewhere (Hogg et al., 2003a)). Infected mice were culled at 6, 24 and 96 h p.i. and compared with age- and sex-matched uninfected control (naïve) mice. Cercarial E/S material (also termed 0–3 h released proteins (0–3hRP)) was collected from live transforming cercariae and concentrated as previously described (Jenkins and Mountford, 2005). An equivalent volume of RPMI medium without parasite material but prepared in the same way (control RPMI (cRPMI)) was used as a negative control for E/S stimulation.

### 2.3 Isolation of epidermal and dermal cells

Pinnae were removed and their thickness quantified using a dial gauge micrometre (Mitutoyo, Japan). Freshly isolated pinnae were then split along the central cartilage and each half portion was floated, dermis side down, on DMEM supplemented with 1% heat inactivated FCS, 50 U/ml of penicillin, 50 μg/ml of streptomycin and 10 mM HEPES (all from Life Technologies, UK; as described previously (Hogg et al., 2003b)) with the addition of 0.4 Wünsch units/ml of Liberase TL enzyme cocktail (Roche, UK) and were incubated at 37 °C for 30 min. Enzymes were neutralised with DMEM/10% FCS/penicillin/streptomycin/HEPES and the epidermal sheet was separated from the dermis before the tissues were separately minced using sterile scissors. Suspensions of epidermal and dermal cells were passed through a cell strainer (BD Biosciences, UK) and resuspended in fresh media prior to enumeration and assessment of viability using Trypan blue dye (Life Technologies).

### 2.4 Flow cytometry

Cell suspensions were washed in cold PBS, centrifuged at 800*g*, resuspended in PBS/0.1% LIVE/DEAD Fixable Viability aqua dye (Invitrogen, UK), and then incubated on ice for 30 min. Cells were washed in fresh PBS/1% FCS and resuspended in 10 μl of goat serum to block non-specific antibody binding for 30 min on ice. Cell surface markers were labelled using cocktails of specific fluorophore-conjugated rat anti-mouse antibodies (eBioscience, UK; Supplementary Table S1) and incubated on ice for 30 min. Cells were finally washed and resuspended in PBS/1% FCS prior to analysis using a Dako Cytomation Flow cytometer (Dako UK Ltd, UK). In some instances, cells were fixed in PBS/1% paraformaldehyde (PFA). Flow cytometry experiments were analysed as proportion and frequency data collated from four independent experiments for naïve, 6 h and 24 h p.i. and three experiments for the 96 h time-point (*n* = 3 mice per time-point per experiment).

### 2.5 Immunofluorescence

Freshly recovered pinnae were fixed in PBS/4% PFA on ice for 30 min. Pinnae were then rinsed with cold PBS and transferred to PBS/15% sucrose for a further 1 h on ice. Fixed pinnae were then embedded in optimal cutting temperature (OCT) medium (Sakura Finetek, Netherlands), and frozen at −80 °C overnight. Frozen pinnae were equilibrated to −20 °C and then cut into 5 μm sections which were affixed to glass microscope slides at room temperature for at least 18 h. All subsequent processing was conducted at room temperature. Sections were simultaneously blocked and permeabilised in PBS/5% goat serum/0.05% saponin (Sigma, UK) for 30 min and then incubated with primary antibodies diluted in PBS/5% goat serum/0.05% saponin for 1 h. After three washes in PBS/0.05% saponin, sections were incubated with fluorophore-conjugated secondary antibodies for 45 min without light exposure (primary and secondary antibodies are listed in Supplementary Table S1). Finally, sections were washed three times in PBS/0.05% saponin, counter-stained with 2 μg/ml of DAPI (Life Technologies) for 5 min and washed two further times in distilled water. Slides were mounted in Prolong Gold AntiFade reagent (Life Technologies) overnight prior to analysis using a Zeiss 710 inverted confocal microscope. All grouped images were analysed using identical acquisition settings in Zeiss ZEN software.

### 2.6 Quantitative PCR (qPCR)

Epidermal tissue for qPCR analysis was immediately transferred to Trizol reagent (Life Technologies) after isolation from the dermis and stored at -80 °C. Total epidermal RNA was extracted using the phenol-chloroform method, treated with TURBO DNase (Life Technologies) to remove contaminating genomic DNA and the resulting RNA concentrations were quantified using a Nanodrop analyser (Thermo Scientific, UK). cDNA was synthesised using the same quantity of RNA from each sample using Superscript II DNA polymerase according to the manufacturer’s instructions (Invitrogen). mRNA expression for genes of interest (*Il1a*, (IL-1α); *Il1b*, (IL-1β); *Il33* (IL-33); *Tslp* (TSLP); *Ccl20* (CCL20, a leukocyte chemoattractant); keratin 6 (*Krt6b*) and the reference gene *Gapdh* (glyceraldehyde 3-phosphate dehydrogenase) was quantified via qPCR conducted using Fast SYBR Green reagents and the StepOnePlus™ Real-Time PCR System (Applied Biosystems, UK). Primer pairs used for gene expression analysis are listed in Supplementary Table S2. Fold-change expression of each gene of interest relative to the naïve group was calculated using the 2^−ΔΔCq^ method. The inverse of 2^−ΔΔCq^ values <1 are reported to reflect decreased gene expression. Gene expression analysis was collated from four independent experiments (*n* = 12 naïve animals; *n* = 7 animals at 6 h; *n* = 6 animals at 24 h; *n* = 12 animals at 96 h p.i.).

### 2.7 Neonatal keratinocyte culture

Three day old male C57BL/6 pups were culled and their skin sterilised in 20% Betadine, before being removed according to previously described protocols (Lichti et al., 2008). Skin was incubated at 4 °C, dermal side down, in 3 ml of cold PBS containing 5 mg/ml of Trypsin (Sigma), 50 U/ml of penicillin, 50 μg/ml of streptomycin and 0.5% Fungizone (i.e. 2.5 μg/ml of amphotericin B and 2.05 μg/ml of sodium deoxycholate; Life Technologies) per mouse overnight. Epidermal sheets were isolated from dermal tissue and minced with scissors in 5 ml of CnT-PR (a low calcium serum-free media formulation designed to promote keratinocyte precursor proliferation; Cell-n-Tech, Caltag Medsystems, UK), passed 10× through a serological pipette, centrifuged at 800*g* and resuspended in CnT-PR. Cells were passed through a cell strainer to remove debris, plated at 2 × 10^6^ viable cells per well in a 24 well tissue culture plate containing CnT-PR and incubated at 37 °C with 5% CO_2_. At days 3–4 after seeding, at which point their morphology and keratin expression patterns most resemble basal keratinocyte precursors (Vollmers et al., 2012; Lamb and Ambler, 2013), keratinocytes were stimulated with 100 μg/ml of cercarial E/S, or an equivalent volume of cRPMI for 6 h or 24 h. IL-1α, IL-1β and TSLP were quantified in cell culture supernatants using commercial ELISA kits according to the manufacturer’s instructions (R&D Systems, UK). Keratinocytes grown on glass coverslips were permeabilised and stained for immunofluorescence analysis as described in Section 2.5.

### 2.8 Statistical analysis

Groups were first compared via one-way ANOVA to identify overall differences due to time-points and, where ANOVAs were significant, comparisons between infected groups and the naïve control group were made via post-hoc unpaired *t*-tests or post-hoc Fisher’s Least Significant Difference tests. Comparisons of cultured keratinocyte supernatant cytokine levels in response to different stimuli were made via un-paired *t*-tests. All analyses were conducted in GraphPad Prism version 6 (GraphPad Software Inc, USA). *P* < 0.05 was considered to be statistically significant.

## 3 Results

### 3.1 Percutaneous exposure to Schistosoma larvae alters the epidermal immune environment

**Fig. 1:**
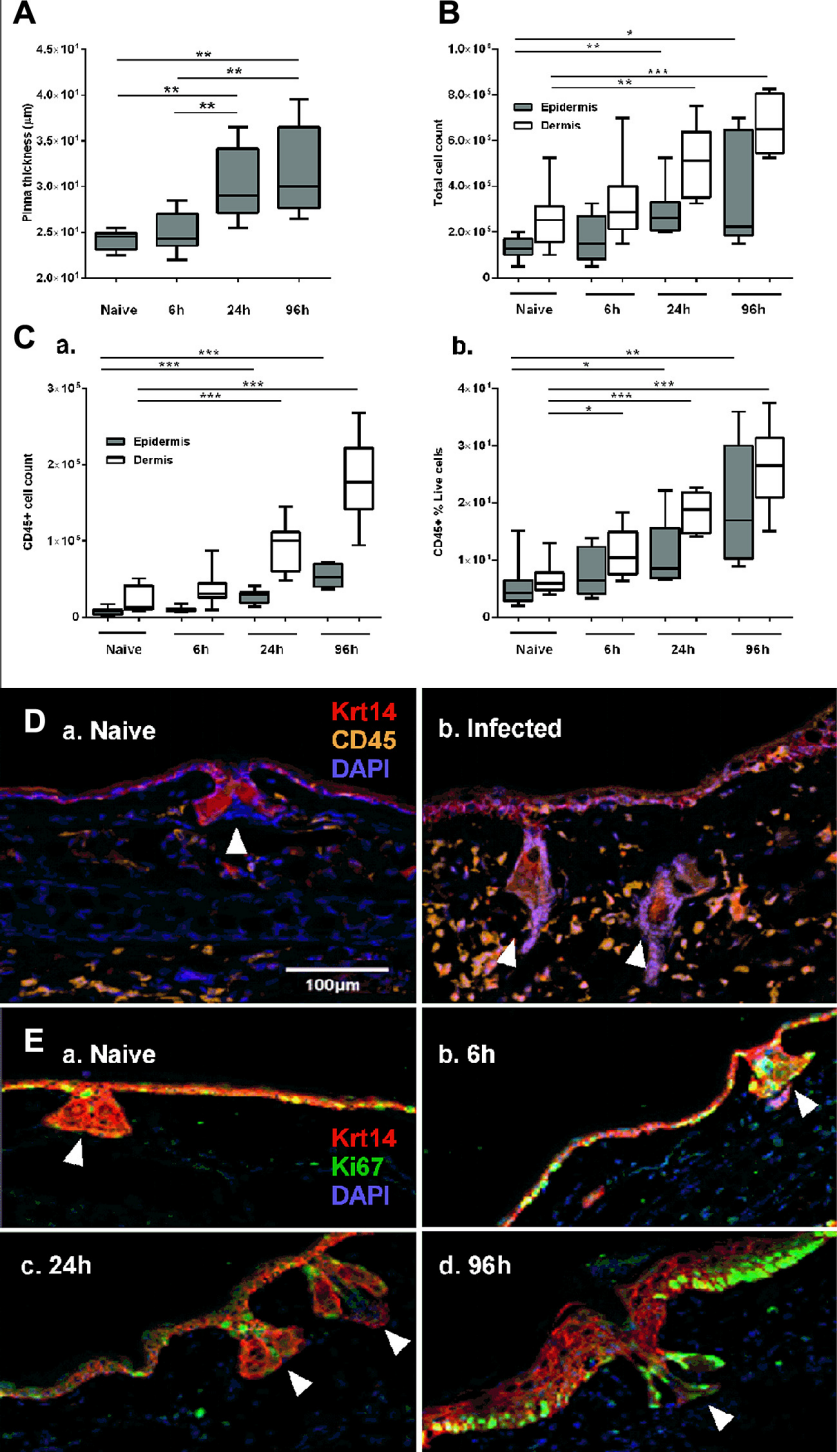
Percutaneous *Schistosoma mansoni* invasion activates cellular responses in the murine epidermis. (A) Pinnae thickness and (B) total cell numbers isolated from naïve and *Schistosoma*-infected pinnae at 6, 24 and 96 h p.i. (C) Number (a) and percentage (b) of CD45^+^ cells in suspensions of whole epidermis (filled) and dermis (open) from naïve and infected animals (*n* = 4 independent experiments, three mice per group for each experiment; box plots indicate median ± 95% confidence interval). Cryosections of pinnae incubated with antibodies specific for (D) basal layer keratin (Krt) 14^+^ keratinocytes and CD45+ cells and (E) the proliferation marker Ki67. White arrows indicate hair follicles and are angled in line with the hair shaft. Microscopy images are representative from at least three independent experiments. Unpaired *t*-tests were performed where ANOVA was significant; ^∗^*P* < 0.05, ^∗∗^*P* < 0.01, ^∗∗∗^*P* < 0.001. Scale bar = 100 μm.

Pinnae of infected mice became visibly inflamed (Fig. 1A), which was accompanied by a corresponding increase in the total number of epidermal and dermal cells recovered from the enzyme-digested tissues (Fig. 1B; epidermis ANOVA *F*: 4.308, *P* = 0.0136, dermis ANOVA *F*: 10.54, *P* < 0.001). In the epidermis there were also significantly higher numbers and percentages of CD45^+^ leukocytes within 24 h of infection than in naïve tissue where resident leukocytes (i.e. γδ T cells and LCs) were a relatively minor population (Fig. 1Ca, Cb; number of CD45^+^ cells: ANOVA *F*: 39.22, *P* < 0.001 and percentages: ANOVA *F*: 6.491, *P* = 0.002). The influx of CD45^+^ cells into the epidermis following infection was also visualised in pinnae cryosections which showed that the frequency of epidermal CD45^+^ cells was greater in 24 h infected animals than in naïve skin (Fig. 1D).

**Supplementary Fig. S1:**
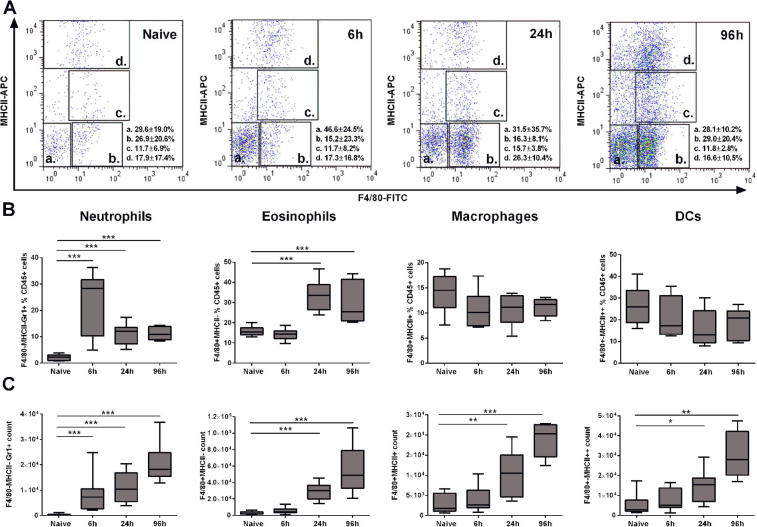
*Schistosoma mansoni* infection elicits leukocyte infiltration of the murine dermis. (A) Flow cytometry gating strategy to identify CD45^+^leukocytes, a) neutrophils F4/80^-^MHCII^-^; Gr1^+^staining used to confirm identity, data not shown), b) eosinophils, (F4/80+MHCII-; Siglec-F+ staining used to confirm identity, data not shown), c) macrophages (F4/80^+^MHCII^+^) and d) dendritic cells (DCs) (F4/80^+/-^MHCII^++^) recovered from the dermis of naïve mice and infected mice at 6, 24 and 96 h p.i. (representative flow cytometry plots from four independent experiments). (B) Median percentages (± 95% confidence interval) and (C) numbers (± 95% confidence interval) of each leukocyte population within the CD45^+^ cell gate. (*n* = 4 independent experiments, three mice per group for each experiment). Unpaired t-tests were performed where ANOVA was significant; ∗*P* < 0.05, ∗∗*P* < 0.01, ∗∗∗*P* < 0.001.

CD45^+^ leukocytes also significantly increased in number and percentage in the dermis (Fig. 1Ca, Cb). Moreover, due to the abundance of CD45^+^ cells in the dermis versus the epidermis, we were able to phenotype sub-types of CD45^+^ dermal cells (Supplementary Fig. S1). The numbers and percentages of F4/80^−^ MHCII^−^ Gr1^+^ neutrophils increased within the first 6 h of infection, whilst F4/80^+^ MHCII^−^ Siglec-F^+^ eosinophils, F4/80^+^ MHCII^+^ macrophages, and F4/80^+^ MHCII^++^ DCs were not significantly higher than those present in naïve dermis until 24 h p.i. (Supplementary Fig. S1).

We hypothesised that changes in dermal and epidermal CD45^+^ leukocyte populations would correspond to activation of keratinocytes, which was evident in infected epidermis as an increased frequency of keratinocyte Ki67^+^ nuclei within the Krt 14^+^ basal keratinocyte regions in both the interfollicular epidermis and hair follicles relative to the homeostatic numbers of proliferating keratinocytes visible in naïve tissue (Fig. 1E). Changes in Ki67 expression were observed throughout the epidermis and across large sections of skin with no evidence of focal changes to indicate specific points of entry by individual cercariae (Fig. 1E).

### 3.2 CD34^+^ keratinocytes expand in the epidermis after percutaneous exposure to Schistosoma larvae

**Fig. 2:**
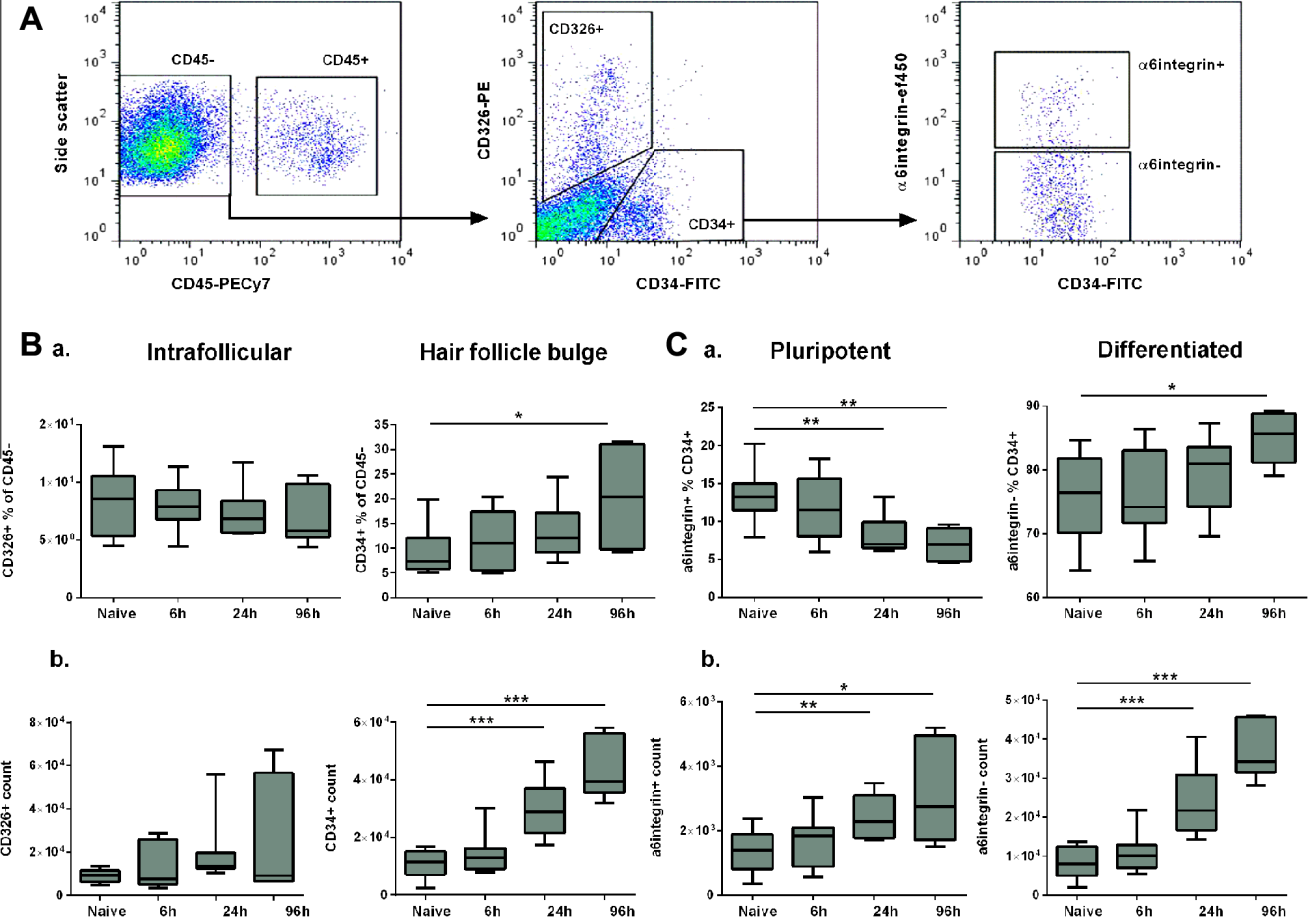
Murine CD34+ epidermal keratinocytes expand and differentiate with time post-*Schistosoma mansoni* infection. (A) Flow cytometry gating strategy to identify CD45^−^ keratinocytes with a CD326^+^ interfollicular and CD34^+^ hair follicle bulge-associated phenotype in epidermal cell suspensions. Surface expression of the pluripotency marker α6integrin is shown within the CD34^+^ population (representative flow cytometry plots from four independent experiments). (B) Proportions (a) and cell numbers (b) of keratinocyte sub-populations within the non-haematopoietic CD45^−^ gate. (C) Proportions (a) and cell numbers (b) of α6integrin^+^ and α6integrin^−^ cells within the CD34^+^ gate (*n* = 4 independent experiments, three mice per group for each experiment; box plots indicate median ± 95% confidence interval). Unpaired *t*-tests were performed where ANOVA was significant; ^∗^*P* < 0.05, ^∗∗^*P* < 0.01, ^∗∗∗^*P* < 0.001.

Given the recently identified diversity within basal keratinocyte populations (Hsu et al., 2011; Jensen et al., 2008; Nagao et al., 2012), the numbers and proportions of keratinocyte sub-types was investigated following infection with *S. mansoni* cercariae. Within the non-haematopoietic (CD45^−^) epidermal keratinocytes, two populations of cells were identified according to expression of the interfollicular keratinocyte marker CD326^+^ (EpCAM) or hair follicle keratinocyte precursor marker CD34^+^ (Fig. 2A) (Jensen et al., 2008; Hsu et al., 2011; Nagao et al., 2012). Following percutaneous exposure to *S. mansoni* cercariae, CD326^+^ keratinocytes in infected epidermis did not differ significantly from naïve samples in either percentage or cell number at any time point (Fig. 2Ba; ANOVA *F*: 0.426, *P* = 0.736 and Fig. 2Bb; ANOVA *F*: 1.457, *P* = 0.249). In contrast, there was a progressive increase in the percentage of CD34^+^ cells with time p.i. (Fig. 2Ba; ANOVA *F*: 3.601, *P* = 0.027), although the increase was not statistically significant until 96 h p.i. The number of CD45^−^ CD326^−^ CD34^+^ hair follicle-associated keratinocytes also significantly increased 24 h after infection and this expansion was sustained at 96 h p.i. (Fig. 2Bb; ANOVA *F*: 23.98, *P* < 0.001).

### 3.3 Expanded CD34^+^ keratinocytes in schistosome-infected skin have a differentiated phenotype

To further investigate the phenotype of the expanding CD34^+^ keratinocyte population, cell surface expression of α6integrin, a cell adhesion molecule associated with pluripotency (Kaur and Li, 2000; Trempus et al., 2003; Muller et al., 2008), was examined. In the CD45^−^ CD326^−^ CD34^+^ cell population both α6integrin^−^ (ANOVA *F*: 27.96, *P* < 0.001) and α6integrin^+^ cells (ANOVA *F*: 4.655, *P* = 0.010) had significantly increased in number by 24 h p.i. (Fig. 2Cb). However, due to the greater increase in the number of α6integrin^−^ relative to α6integrin^+^ cells, the percentage of pluripotent CD34^+^ α6integrin^+^ cells significantly declined with time p.i. relative to naïve skin (Fig. 2Ca; ANOVA *F*: 5.960, *P* = 0.003), suggesting that the expanding population of CD34^+^ keratinocytes developed a differentiated surface phenotype with time post-schistosome infection. Proportions of α6integrin^−^ cells tended to increase p.i. relative to naïve skin, however this difference was not statistically significant until 96 h p.i. (Fig. 2Ca; ANOVA *F*:3.154, *P* = 0.042).

### 3.4 Schistosome-responsive CD34^+^ keratinocytes are associated with the basal bulge of hair follicles

**Fig. 3:**
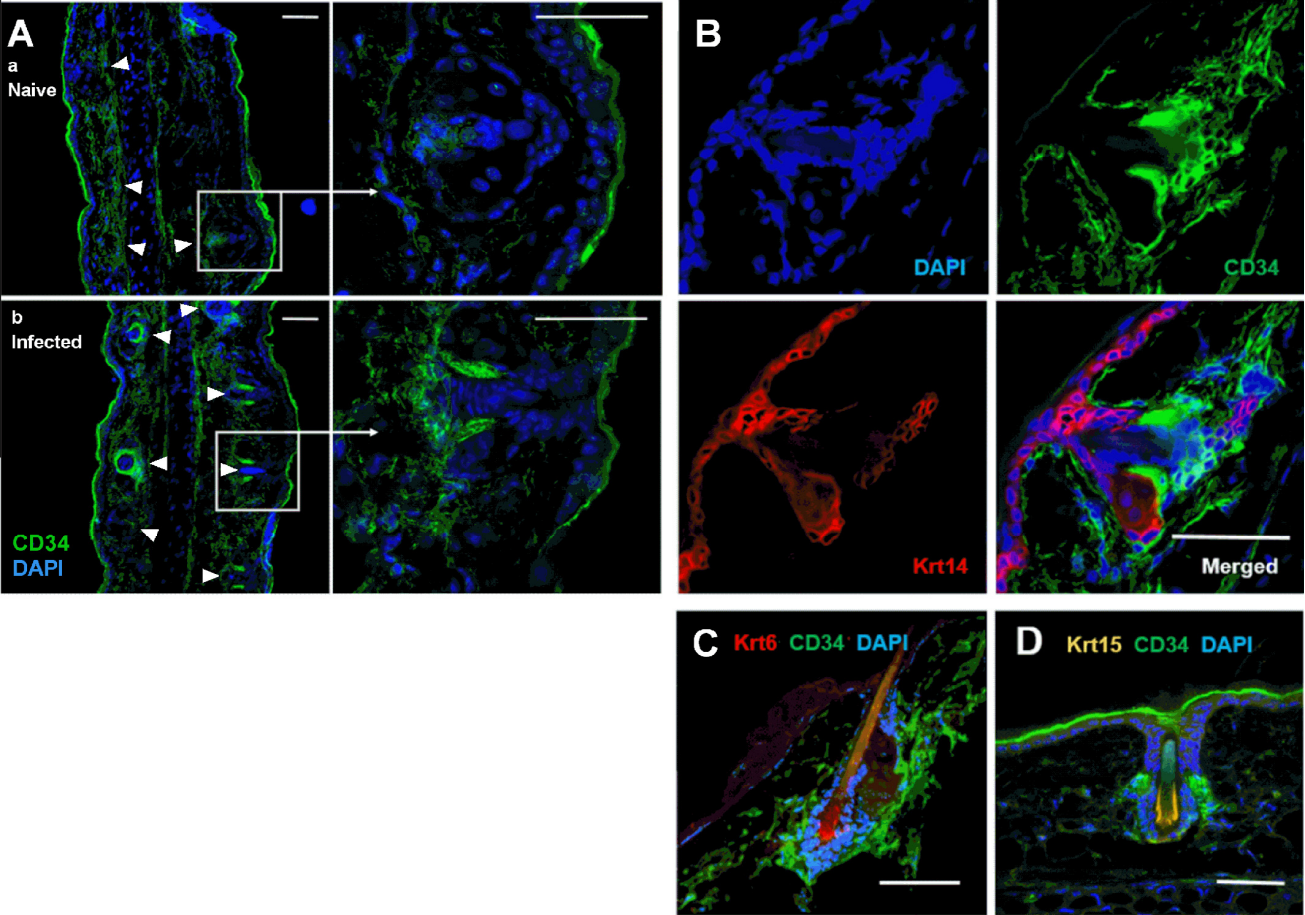
Expanded CD34+ keratinocytes in *Schistosoma mansoni*-infected mouse skin are located in the basal bulge of the hair follicle and are negative for mature keratins (Krt). (A) Cryosections from naïve (a) and infected (b; 24 h p.i.) pinnae labelled with antibodies specific for CD34. Image at 20× magnification (white arrows indicate hair follicles), and 63× magnification (white box, inset) showing individual hair follicles. (B) Individual and merged fluorescence channel views of an infected pinna cryosection labelled for CD34 and Krt 14, and counterstained with DAPI. Pinnae showing cells positive for (C) CD34 and Krt 6, and (D) CD34 and Krt 15. A and B are representative images from at least three independent experiments; C and D are representative images from at least two independent experiments. Scale bar = 50 μm.

In unwounded skin, pluripotent keratinocyte precursor cells are localised within the outer root sheath of the basal bulge of the hair follicle (Hsu et al., 2011) and this niche becomes mobilised to initiate wound healing responses upon mechanical damage (Plikus et al., 2012). Comparison of pinnae cryosections showed a marked increase in the intensity of labelling with CD34 specific antibodies in infected relative to naïve epidermis, and the localisation of CD34^bright^ areas of cryosections in and around hair follicle structures (Fig. 3A). At higher magnification it is evident that CD34^bright^ areas are adjacent to the base of hair shafts in the basal bulge region (Fig. 3Ab). Antibody labelling for mature Krts 6, 14 and 15 showed that CD34^+^ cells are in close apposition to, but outside of, Krt 6^+^, Krt 14^+^ and Krt 15^+^ regions (Fig. 3B–D), and thus correspond to the previously described outer hair follicle bulge stem cells (or keratinocyte precursor) phenotype, rather than mature basal epidermis (Krt 14^+^), or the Krt 6^+^ and Krt 15^+^ cells of the inner hair follicle bulge (Hsu et al., 2011).

### 3.5 Infection induces transient up-regulation of pro-inflammatory markers in the epidermis

**Fig. 4:**
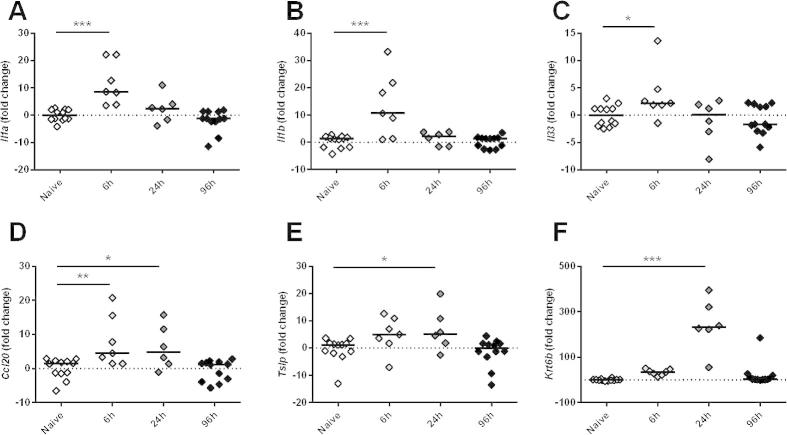
Percutaneous infection by *Schistosoma mansoni* larvae elicits transient up-regulation of stress-associated genes in the murine epidermis. Quantitative PCR analysis of mRNA expression in epidermal tissue isolated from the pinnae of naïve and schistosome-infected mice. Fold change values for infected groups relative to the naïve group calculated for (A) *Il1a* (IL-1α), (B) *Il1b* (IL-1β), (C) *Il33* (IL-33), (D) *Ccl20* (CCL20), (E) *Tslp* (thymic stromal lymphopoietin), and (F) *Krt6b* (keratin 6b) all normalised to *Gapdh* (glyceraldehyde 3-phosphate dehydrogenase) expression in the same samples (2^−ΔΔCq^ method). Each data point represents gene expression analysis for one mouse (i.e. epidermal tissue combined from two pinnae) and data is combined from two (6 h and 24 h) or four independent experiments (naïve and 96 h). Post-hoc Fisher’s Least Significant Difference tests were used where ANOVA was significant; ^∗^*P* < 0.05, ^∗∗^*P* < 0.01, ^∗∗∗^*P* < 0.001.

To assess whether the epidermal changes that we had observed equated to immunologically relevant changes in gene expression, cytokine (*Il1a*, *Il1b*, *Il33* and *Tslp*), the leukocyte chemoattractant *Ccl20* and the stress-associated activation marker *Krt6b* gene expression were assayed in epidermal cell suspensions. The transcript levels for *Il1a* (ANOVA: *F*: 13.29, *P* < 0.001), *Il1b* (ANOVA: *F*: 11.37, *P* < 0.001), *Il33* (ANOVA: *F*: 3.452, *P* = 0.028) and *Ccl20* (ANOVA: *F*: 6.824, *P* = 0.001) were all elevated within 6 h of infection relative to naïve pinnae (Fig. 4A–D), but *Il1a*, *Il1b* and *Il33* had declined to baseline levels by 24 h (Fig. 4A–C). Conversely, *Tslp* (ANOVA: *F*: 4.015, *P* = 0.015) and *Krt6b* (ANOVA: *F*: 30.20, *P* < 0.001) were not significantly higher until 24 h p.i. (Fig. 4E, F). By 96 h p.i., all six genes were expressed at levels equivalent to the naive group.

### 3.6 Keratinocyte precursors produce pro-inflammatory cytokines in response to schistosome E/S in vitro

**Fig. 5:**
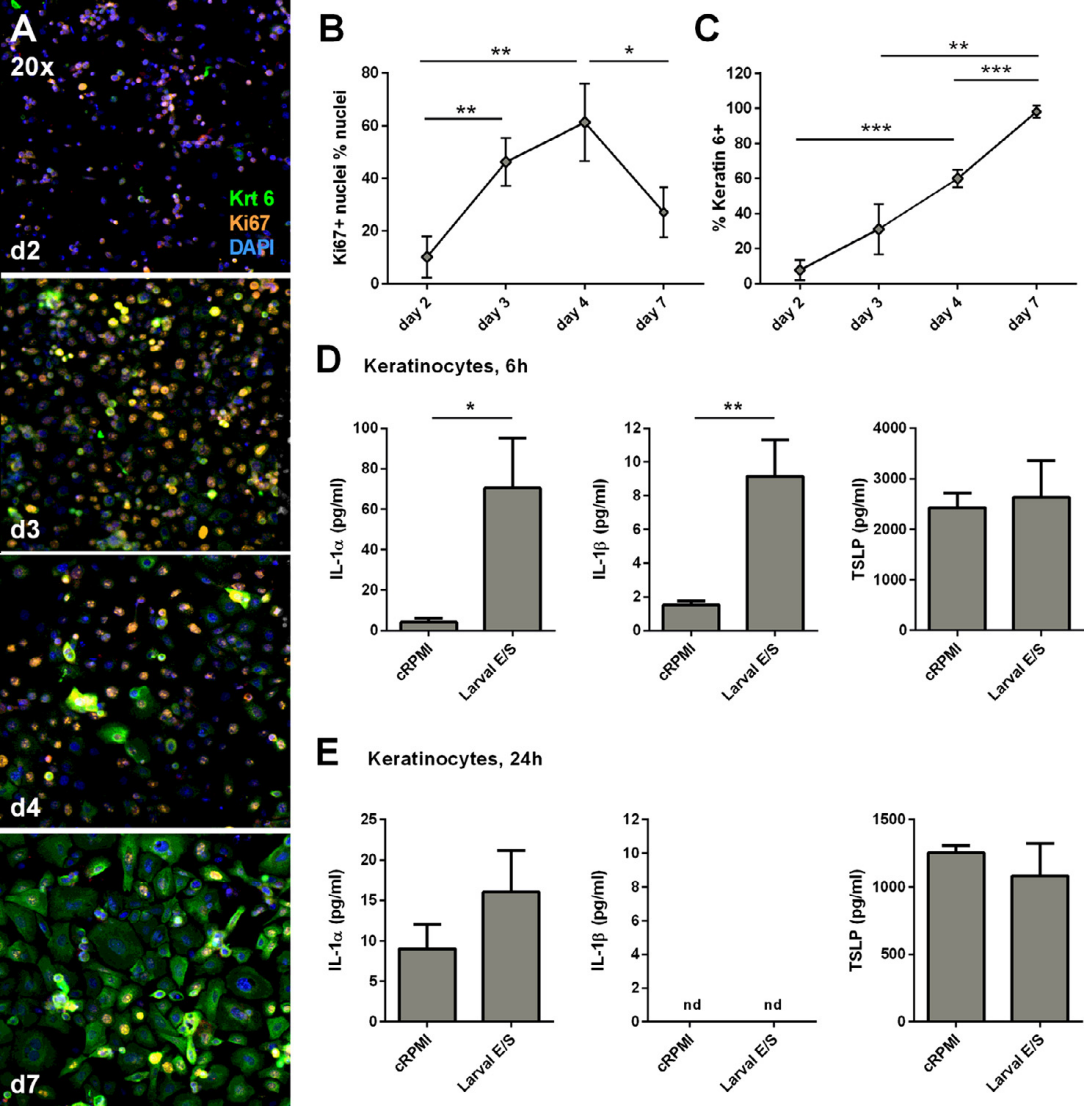
Keratinocyte precursors produce transient pro-inflammatory cytokine responses to *Schistosoma mansoni* larval excretory/secretory antigens in vitro. (A) Representative confocal micrographs of keratinocyte monolayers at days (d) 2, 3, 4 and 7 post-seeding, labelled with antibodies specific for mature keratin (Krt) 6 and Ki67, and counter-stained with DAPI (20× magnification). Mean percentage of (B) Ki67^+^ nuclei from total nuclei and (C) Krt 6+ cells from total cells in keratinocyte cultures at days 2, 3, 4 and 7 post-seeding. (Error bars: S.D. determined from three non-overlapping image frames per time point). Keratinocyte precursors were stimulated with 100 μg/ml of larval excretory/secretory antigen, or control RPMI medium in the proliferative (undifferentiated) stage of the growth cycle (days 3–4 post-seeding). Quantification of IL-1α, IL-1β and thymic stromal lymphopoietin levels in keratinocyte precursor culture supernatants stimulated for (D) 6 h and (E) 24 h with larval excretory/secretory or control RPMI medium (*n* = 3 culture wells from a single experiment; data is representative of three independent experiments). Unpaired *t*-test; ^∗^*P* < 0.05, ^∗∗^*P* < 0.01. Figure legend: 'nd - not detectable'.

Innate cytokines are key initiators of pro-inflammatory responses, which trigger activation of keratinocytes (Freedberg et al., 2001), cause infiltration of professional immune cells, and escalate the production of pro-inflammatory signals in cutaneous tissue (Barker, 2004). Since keratinocyte-specific cytokine production is difficult to distinguish from that of surrounding leukocytes and dermal fibroblasts in whole pinnae tissue, keratinocyte precursor monolayers were used to investigate keratinocyte responses to schistosome cercarial E/S in vitro. To model the keratinocyte precursor differentiation state, Ki67 and Krt 6 expression was assessed by immunohistochemistry in cultured cells (Fig. 5A). Cells at days 3 and 4 post-seeding were in a more proliferative state than at earlier or later time points (i.e. highest percentage of Ki67^+^ nuclei; Fig. 5B, ANOVA: *F*: 13.28, *P* = 0.002) with relatively low expression of the activation marker Krt 6 (Fig. 5C, *F*: 65.74, *P* < 0.001) compared with later time points. Thus keratinocyte monolayers at days 3–4 post-seeding were most phenotypically similar to Krt 6- keratinocyte precursors (Vollmers et al., 2012; Lamb and Ambler, 2013). By day 7 post-seeding, cells displayed a differentiated phenotype with a reduced frequency of Ki67^+^ nuclei (Fig. 5B), higher expression of Krt 6 (Fig. 5C) and the formation of three-dimensional strata (data not shown).

Keratinocyte precursors stimulated for 6 h with schistosome E/S on day 3 or 4 post-seeding relative to parallel cultures stimulated with cRPMI, had significantly elevated levels of IL-1α and IL-1β in culture supernatants (Fig. 5D). There was no significant difference in the level of TSLP detected at 6 h between E/S and cRPMI stimulated cultures (Fig. 5D). However, the increase in IL-1α and IL-1β production was transient since after 24 h of antigen stimulation, levels were equivalent to those present in cultures incubated with cRPMI (Fig. 5E).

## 4 Discussion

Epidermal keratinocytes are known to be rapidly activated by mechanical wounding and their mobilisation is an essential part of repairing damaged skin (Blanpain and Fuchs, 2009; Plikus et al., 2012). In schistosomiasis, larval parasites must breach the epidermal barrier in order to reach the dermis and continue onward migration to the vasculature where egg production by mature parasites leads to disease pathology (Wheater and Wilson, 1979). However, immunologically relevant changes in the epidermis during the early phase of schistosome infection have not been previously characterised. Here we demonstrate for the first known time that epidermal keratinocytes respond to schistosome infection by differentiating and increasing proliferation rates, and that larval E/S antigens promote pro-inflammatory cytokine secretion by keratinocyte precursors in vitro. Our observation that keratinocyte precursors in the outer region of the basal bulge of hair follicles are specifically expanded following *Schistosoma* challenge mimics the mobilisation of this usually quiescent stem cell niche during wound healing (Blanpain and Fuchs, 2009; Hsu and Fuchs, 2012).

Detailed characterisation of the distinct cellular niches within the hair follicle has demonstrated compartmentalisation of stem-like cells in the basal bulge of the hair follicle (Blanpain and Fuchs, 2009; Hsu et al., 2011), which can be further subdivided into an inner root sheath layer of Krt 6^+^ cells that restrict expansion of slow cycling pluripotent CD34^+^ cells in the outer root sheath during the resting phase of the hair cycle (Hsu et al., 2011). Krt 6 is down-regulated in pluripotent stem cells but can be induced in response to pro-inflammatory signals and thus is both a phenotypic marker of keratinocyte precursor sub-sets and an activation marker in the interfollicular epidermis (Freedberg et al., 2001; Hsu et al., 2011; Vollmers et al., 2012). Thus, in naïve mice the epidermis is in a ‘steady state’ with a small population of CD34^+^ basal bulge cells which have a pluripotent (Krt 6- α6integrin^+^) phenotype, low levels of proliferation in the hair follicle and low levels of activation and proliferation in the interfollicular epidermis. Following *Schistosoma* infection however, we show that the CD34+ cell population both expands and changes in phenotype to become more differentiated (α6integrin^−^) with time p.i. relative to naïve epidermis. These quantitative changes in the CD34^+^ keratinocytes were also visualised in pinna cryosections, where CD34^+^ hair follicle associated keratinocyte precursor cells were identified as being distinct from Krt 6^+^ suprabasal cells (Hsu et al., 2011). This was accompanied by an increase in the frequency of proliferating basal keratinocytes (Krt 14^+^ Ki67^+^), both in the hair follicle and interfollicular epidermis, and an increase in *Krt6b* expression in whole epidermal tissue, indicating that infection initiates the keratinocyte activation cycle (Freedberg et al., 2001). The lack of expression of mature keratins by the expanding CD34^+^ hair follicle-associated keratinocytes, particularly Krt 14 which is highly expressed by keratinocytes throughout the basal layer of the epidermis, further highlights the distinction between the cells that expand in response to infection and the more differentiated keratinocyte sub-types forming the basal and supra-basal epidermal layers (Blanpain and Fuchs, 2009; Hsu et al., 2011; Hsu and Fuchs, 2012).

The putative role of hair follicle bulge stem cells is to reconstitute damaged interfollicular epidermis following wounding (Blanpain and Fuchs, 2009), as occurs following infection with *Schistosoma* cercariae when structural skin proteins are targeted by proteolytic enzymes in cercarial E/S. Indeed, previous proteomic analyses of human skin exposed to *S. mansoni* revealed that enzymes within cercarial E/S directly target keratinocyte (e.g. krt 9) and extracellular matrix components (e.g. collagen and fibronectin) (Ingram et al., 2011), indicating that wounding of the epidermis is a necessary part of skin invasion. It has also been suggested that hair follicles are directly targeted as a point of entry by *S. mansoni* cercariae, which have been visualised in the base of hair follicles in photomicrographs of infected murine skin (Standen, 1953) and observed to enter live human skin via hair follicles in ∼4% of penetration events (Haas and Haeberlein, 2009). However, this appears to be a relatively minor occurrence relative to penetration at interfollicular sites (Haas and Haeberlein, 2009) and was not a phenomenon observed during the current study nor in a previous live imaging study by our group (Paveley et al., 2009).

A range of soluble mediators released by keratinocytes initiate pro-inflammatory immune responses to skin wounding (Barker, 2004). This is characterised by an ‘initiation phase’ involving IL-1α and IL-1β release from activated keratinocytes, an ‘amplification phase’ driven by cytokines derived from infiltrating leucocytes and activated dermal fibroblasts, and a ‘resolution phase’ involving immunoregulatory cytokines (Barker, 2004). Our study demonstrates that stimulation of precursor keratinocytes in vitro with schistosome E/S antigens elicited production of IL-1α and IL-1β which corresponds to an early (6 h) increase in the expression of *Il1a* and *Il1b* mRNA transcripts in whole epidermal tissue. IL-1α and IL-1β act as molecular triggers for dermal fibroblast responses, including the release of keratinocyte growth factors that escalate epidermal activation (Maas-Szabowski et al., 2000; Canady et al., 2013). Production of IL-1α, IL-1β and the soluble IL-1α receptor in response to *Schistosoma* E/S has been previously shown in neonatal human keratinocytes in vitro (Ramaswamy et al., 1995a), however, unlike our study, the keratinocyte differentiation state was not determined and it is therefore unclear which epidermal niche this earlier study was modelling. Keratinocyte-derived pro-inflammatory mediators are also elicited in response to other cutaneous pathogens. For example, *S. scabiei* extracts and whole mites elicit IL-1α and IL-1β production by human skin equivalents (Arlian et al., 1996), as well as the production of growth-related oncogene alpha, transforming growth factor alpha, and cutaneous T-cell attracting chemokine by keratinocytes (Mullins et al., 2009).

Since invasion by schistosome cercariae comprises both mechanical damage and exposure of skin cells to pathogen-associated molecular patterns (PAMPs), including those found in cercarial E/S (Paveley et al., 2009, 2011), it was not possible to distinguish the relative contributions of these two stimuli to keratinocyte activation in vivo. The specific localisation of the changes that we observed in basal bulge keratinocytes shows a strong concordance with observations following wounding (Hsu et al., 2011). However, secretion of IL-1α and IL-1β by primary keratinocyte monolayers in response to cercarial E/S stimulation in vitro in the absence of mechanical wounding suggests that keratinocytes can also directly sense schistosome antigens. Given the intimate associations between the immunobiology of wound healing and helminth infection (Gause et al., 2013), it seems likely that both pathways may play a role in keratinocyte responses to cutaneous schistosomiasis. Toll like receptors (TLRs) are candidate pattern recognition receptors (PRRs) for the recognition of PAMPs within cercarial E/S since they are constitutively expressed by keratinocytes (Baker et al., 2003). TLRs are also known to be involved in the recognition of larval E/S by APCs (Jenkins et al., 2005a,b), alongside phagocytic receptors such as the mannose receptor (Paveley et al., 2011). Further studies are required to characterise both the schistosome antigens that drive keratinocyte activation in the epidermis and the PRRs to which they ligate.

The rapid cellular changes in the dermis during the first 4 days post-*Schistosoma* infection, observed here and in previous studies (Jenkins et al., 2005b; Paveley et al., 2009; Cook et al., 2011), raises an important question as to how keratinocyte activation in the epidermis influences down-stream mobilisation of dermal APC and the subsequent development of acquired immune responses. Within 24 h of infection, expression of *Il33*, *Tslp* and *Ccl20* transcripts were elevated in infected epidermis relative to naïve skin, indicating that the epidermis is a source of stimuli for APCs and T cells. For example, IL-33 and TSLP promote both the development of a wound healing phenotype in dermal APCs and Th2 polarisation in skin-draining lymph nodes (Cook et al., 2011; Gause et al., 2013), whilst CCL20 is a chemoattractant for CCR6^+^ T cells and immature DCs in murine models of psoriasis (Harper et al., 2009), and thus may be involved in driving these processes following exposure to cercariae. In addition, hair follicle keratinocytes specifically secrete chemokines required to recruit LC precursors to the epidermis within 24 h of LC depletion in mice (Nagao et al., 2012), providing further evidence that these cells promote leukocyte chemotaxis. Due to the much larger number of cells that could be isolated from the dermal tissue relative to the epidermis, we were able to characterise the cell sub-types in the dermal CD45+ infiltrate and demonstrate differences in the timing at which neutrophils, eosinophils, macrophages and DCs are recruited to the dermis. Our observations in whole dermal tissue corroborate previous observations in murine dermal exudate cells (DEC) isolated from schistosome infected pinnae (Cook et al., 2011) and suggest that cutaneous chemotactic signals change with time p.i. Temporal variation in the expression of soluble mediators in whole epidermis suggest that keratinocytes may contribute to this process, however a more detailed characterisation of the CD45+ epidermal infiltrate and leukocyte chemotactic factors derived specifically from keratinocytes in vivo is required to confirm this hypothesis. Since the up-regulation of keratinocyte cytokine and chemokine genes returned to levels equivalent to naïve skin within 96 h, and within 24 h for keratinocyte precursor cultures, it is possible that these mediators are primarily involved in the ‘initiation’ and ‘amplification’ phases of wound-healing type responses to *Schistosoma* infection rather than dominating the ‘resolution’ phase (Barker, 2004).

Collectively, our data demonstrate that percutaneous infection by *S. mansoni* activates the skin stem cell niche, expanding the population of differentiated and proliferating keratinocyte precursors in the hair follicle basal bulge in a manner akin to that observed in studies of mechanical wounding (Blanpain and Fuchs, 2009; Hsu and Fuchs, 2012). Our in vitro observations suggest that keratinocyte precursor activation by *Schistosoma* infection is at least partly related to direct sensing of schistosome PAMPs present in cercarial E/S material. The activation of keratinocytes and the soluble pro-inflammatory mediators that they release may contribute to the infiltration of APCs into the dermis, observed during the first 4 days after a single infection and previously demonstrated in the dermis of repeatedly infected animals (Cook et al., 2011). These insights into the role of keratinocytes and the epidermis more broadly contribute to a more holistic view of how immune responses develop in schistosome-infected skin.
