# Supplementary material for: Epidermal keratinocytes initiate wound healing and pro-inflammatory immune responses following percutaneous schistosome infection
Source: Int J Parasitol. 2015 Mar;45(4):215–24. doi: 10.1016/j.ijpara.2014.11.002 (PMC4365920; doi:10.1016/j.ijpara.2014.11.002)
Supplement: Supplementary Tables [file mmc1.docx]

**Supplementary Table S1.** Antibodies for flow cytometry and immunohistochemistry in this study.

| **Species** | **Isotype^a^** | **Specificity** | **Clone** | **Fluorophore** | **Manufacturer** |
| --- | --- | --- | --- | --- | --- |
| **Epidermal cells^b^** | | | | | |
| Rat | IgG2a, k | CD49f  (α6 integrin) | Monoclonal (GoH3) | eFluor® 450^c^ | eBioscience Inc. |
| Rat | IgG2a, k | CD34 | Monoclonal (RAM34) | FITC | eBioscience Inc. |
| Rat | IgG2a, k | CD326  (EpCAM) | Monoclonal (G8.8) | PE | eBioscience Inc. |
| Rate | IgG2b, k | CD45 | Monoclonal (30-F11) | PE-Cy7 | eBioscience Inc. |
| **Dermal cells^b^** | | | | | |
| Rat | IgG2b, k | CD45 | Monoclonal (30-F11) | eFluor® 450^c^ | eBioscience Inc. |
| Rat | IgG2a, k | F4/80 | Monoclonal (BM8) | FITC | eBioscience Inc. |
| Rat | IgG2a, k | Siglec-F | Monoclonal (E50-2440) | PE | BD Biosciences |
| Rat | IgG2b, k | Ly-6G (Gr-1) | Monoclonal (RB6-8C5) | PE-Cy7 | eBioscience Inc. |
| Rat | IgG2b, k | MHC Class II  (I-A/I-E) | Monoclonal (M5/114.15.2) | APC | eBioscience Inc. |
| **Pinna cryosections^d^** | | | | | |
| Chicken | IgY | Keratin 14 | Polyclonal | none | Covance Inc. |
| Chicken | IgY | Keratin 15 | Polyclonal | none | Covance Inc. |
| Rabbit | IgG | Keratin 6 | Polyclonal | none | Covance Inc. |
| Rat | IgG2a, k | CD34 | Monoclonal (RAM34) | FITC | eBioscience Inc. |
| Rat | IgG2a, k | Ki67 | Monoclonal (SolA15) | PE-Cy7 | eBioscience Inc. |
| Goat | IgG | Chicken IgG^e^ | Polyclonal | Alexa Fluor® 633^f^ | Life Technologies |
| Goat | IgG | Rabbit IgG^e^ | Polyclonal | Alexa Fluor® 488^f^ | Life Technologies |
| Goat | IgG | Rabbit IgG^e^ | Polyclonal | Alexa Fluor® 546^f^ | Life Technologies |
| Goat | IgG | Rat IgG^e^ | Polyclonal | Alexa Fluor® 488^f^ | Life Technologies |
| Goat | IgG | Rat IgG^e^ | Polyclonal | Alexa Fluor® 546^f^ | Life Technologies |

^a^Isotype control antibodies were raised in the same species, conjugated to the same fluorophore and obtained from the same manufacturer as their corresponding marker-specific antibodies

^b^Antibodies used for flow cytometry analysis

^c^eFluor^®^ fluorophores are registered to eBioscience

^d^Antibodies used for immunohistochemical analysis

^e^Antibodies are specific to both heavy and light chains of IgG

^f^Alexa Fluor^®^ fluorophores are registered to Molecular Probes

PE, Phycoerythrin; Cy7, Cyanine7; APC, Allophycocyanin.

**Supplementary Table S2.** Primer pairs used for gene expression analysis of mouse epidermal genes.

| **Gene** | **Forward Primer** | **Reverse Primer** |
| --- | --- | --- |
| ***Gapdh^a^*** | 5’–CCATGTTTGTGAGGGTGTG–3’ | 5’–CCTTCCACAATGCCAAAGTT–3’ |
| ***Il1a^a^*** | 5’–GGGTGACAGTATCAGCAACG–3’ | 5’–TCTGGTAGGTGTAAGGTGCT–3’ |
| ***Il1b^a^*** | 5’–TACAGGCTCCGAGATGAACA–3’ | 5’–AGGCCACAGGTATTTTGTCG–3’ |
| ***Tslp^a^*** | 5’–AGTTCGAGCAAATCGAGGAC–3’ | 5’–TGTGCCATTTCCTGAGTACC–3’ |
| ***Ccl20^a^*** | 5’–GCAAGCAACTACGACTGTTG–3’ | 5’–TTAGGCTGAGGAGGTTCACA–3’ |
| ***Il33*^b^** | 5’–GATGGGAAGAAGCTGATGGTG–3’ | 5’–TTGTGAAGGACGAAGAAGGC–3’ |
| ***Krt6b^a^*** | 5’–TGCCTGGTAAAGTGTGTGTC–3’ | 5’–AAACAAAGAGCAGAGATGGCA–3’ |

^a^Primer pairs were designed using Primer3Plus (Rozen and Skaletsky, 2000) and cross-checked using OligoAnalyzer version 3.1 (Integrated DNA Technologies Inc., USA) and Primer BLAST (National Center for Biotechnology Information, USA) **^b^**Primer pair from Zhao and Hu (2012).

*Gapdh,* glyceraldehyde 3-phosphate dehydrogenase; *Il1a,* IL-1α; *Il1b,* IL-1β; *Tslp,* thymic stromal lymphopoietin; *Ccl20, CCL20 (*leukocyte chemoattractant); *Il33,* IL-33; *Krt6b,* keratin 6b.

**References**

Rozen, S., Skaletsky, H., 2000. Primer3 on the WWW for general users and for biologist programmers. Meth. Mol. Biol. 132, 365 - 386.

Zhao, W.-H., Hu, Z.-Q., 2012. Up-regulation of IL-33 expression in various types of murine cells by IL-3 and IL-4. Cytokine 58, 267 - 273.
